# Adhesion of *Streptococcus mitis* and *Actinomyces oris* in co-culture to machined and anodized titanium surfaces as affected by atmosphere and pH

**DOI:** 10.1186/1472-6831-13-4

**Published:** 2013-01-08

**Authors:** Josefin Seth Caous, Maria Lövenklev, Jenny Fäldt, Maud Langton

**Affiliations:** 1 Department of Chemical and Biological Engineering, Chalmers University of Technology, Gothenburg, SE-402 29, Sweden; 2 SIK, The Swedish Institute for Food and Biotechnology, P.O. Box 5401, Gothenburg, SE-402 29, Sweden; 3 Nobel Biocare AB, P.O. Box 5190, Gothenburg, SE-402 26, Sweden; 4 Department of Food Science, SLU- Swedish University of Agricultural Sciences, PO Box 7051, Uppsala, SE-756 45, Sweden

**Keywords:** Bacterial adhesion, Dental implants, Peri-implant disease, Confocal laser scanning microscopy

## Abstract

**Background:**

With the rising demand for osseointegrated titanium implants for replacing missing teeth, often in patients with a history of periodontitis, implant-related infections have become an issue of growing concern. Novel methods for treating and preventing implant-associated infections are urgently needed. The aim of this study was to investigate if different pH, atmosphere and surface properties could restrict bacterial adhesion to titanium surfaces used in dental implants.

**Methods:**

Titanium discs with machined or anodized (TiUnite™) surface were incubated with a co-culture of *Streptococcus mitis* and *Actinomyces oris* (early colonizers of oral surfaces) at pH 5.0, 7.0 and 9.0 at aerobic or anaerobic atmosphere. The adhesion was analysed by counting colony forming (CFU) units on agar and by confocal laser scanning microscopy (CLSM).

**Results:**

The CFU analysis showed that a pH of 5.0 was found to significantly decrease the adhesion of *S. mitis,* and an aerobic atmosphere, the adhesion of *A. oris*. *S. mitis* was found in significantly less amounts on the anodized surface than the machined surface, while *A. oris* was found in equal amounts on both surfaces. The CLSM analysis confirmed the results from the CFU count and provided additional information on how the two oral commensal species adhered to the surfaces: mainly in dispersed clusters oriented with the groves of the machined surface and the pores of the anodized surface.

**Conclusions:**

Bacterial adhesion by *S. mitis* and *A. oris* can be restricted by acidic pH and aerobic atmosphere. The anodized surface reduced the adhesion of *S. mitis* compared to the machined surface; while *A. oris* adhered equally well to the pores of the anodized surface and to the grooves of the machined surface. It is difficult to transfer these results directly into a clinical situation. However, it is worth further investigating these findings from an in vitro perspective, as well as clinically, to gain more knowledge of the effects acid pH and aerobic atmosphere have on initial bacterial adhesion.

## Background

Advances in dental implantology during the past 20 years have made it possible to provide a greater number of patients with successfully osseointegrated dental implants. [1,2]. However, with the shift from mainly treating fully edentulous patients to patients missing one or a few teeth, often due to periodontitis, implant-related bacterial infections have become an issue of increasing concern [3-5]. Bacteria colonizing the periodontal pockets can spread to the peri-implant tissue and exposed implant part, and may initiate an inflammatory response in the peri-implant tissue [6]. An inflammation in the soft tissue surrounding the implant can disturb the tight connection between the mucosa and the implant abutment. This will enable bacterial adhesion to the smooth surface of the abutment and, if exposed, to the rough surface of the implant [7,8]. If not successfully treated, the inflammation may eventually lead to degradation of the implant-supporting bone, resulting in loss of the implant. Treatment of peri-implant disease today often includes thorough mechanical cleaning of the implant surface and the use of systemic antibiotics, however, there is no guarantee of a successful outcome [9,10].

The initial adherence of bacteria to exposed implant parts will be influenced by variations in the oral environment. The pH and level of oxygen in the oral cavity vary depending on a number of factors, including food intake, oral health and location in the mouth, i.e. air exposed tooth surface vs. the oxygen deprived gingival pocket. [11]. Oral bacteria, such as *Streptococcus mitis* and *Actinomyces oris* (formely *naeslundii* genotype II), are normally found in the air-exposed margin of the gingival crevice, and are continuously flushed by saliva with a neutral pH of 7. However, with the increase in bacterial load the local environment becomes more acidic as a result of bacterial production of lactic acid [12]. Furthermore, inflammation in the implant-surrounding soft tissue leads to increased production of the slightly alkaline gingival crevice fluid [13], and a deepening of the peri-implant pocket [10,14]. With the deepening of the gingival pocket, the oxygen saturation of the crevice fluid decreases [15]. Many of the studies reported in the literature have been focused on the effect of acidification on the caries-causing *Streptococcus mutans*[16,17] ,and on its adhesion to hydroxyapatite, a bioceramic similar to the mineral component of bone and teeth [18]. An acidic pH of 4.5 has been shown to reduce the adhesion of *S. mutans* to hydroxyapatite, while a pH of 6.0 has been reported to have a similar effect on the ability of *A. oris* to adhere [19]. However, no extended research has been conducted on the effect of acidification on bacterial adhesion to the surface of titanium implants and abutments, nor has the effect of alkaline pH on bacterial adhesion been thoroughly investigated, although many antimicrobial irrigation solutions and gels have an alkaline pH [20,21]. Furthermore, most investigations have been performed using a monoculture. Considering the complexity of the microbial community of the oral cavity, the use of two bacteria in a co-culture could provide additional important information.

In order to prevent implant-related infections and to improve the treatment options, more knowledge about bacterial adhesion to implant surfaces, e.g. the influence of surface properties and environmental conditions, is crucial. The aim of this study was to investigate the effect of pH 5.0, pH 7.0 and 9.0 in combination with aerobic or anaerobic atmosphere, on the initial bacterial adhesion of a co-culture of *S. mitis* and *A. oris* to both machined and anodized titanium surfaces; and to analyse if the characteristics of the implant surface affects the initial bacterial adherence. *S. mitis* and *A. oris* are both facultative anaerobes that can survive in an aerobic environment, albeit they have a greater growth potential under anaerobic conditions. Our hypothesis is therefore, that bacterial adhesion is restricted by an aerobic environment and by both acidic and alkaline pH. Furthermore, while rough surfaces usually facilitate bacterial adherence, the anodized surface has a titanium oxide layer, mainly consistent of the crystalline phase anatase, which is known to have antimicrobial properties [22,23]. Thus, we further hypothesize that less bacteria should adhere to the anodized surface than to the machined titanium surface.

## Methods

### Titanium discs

**Figure 1:**
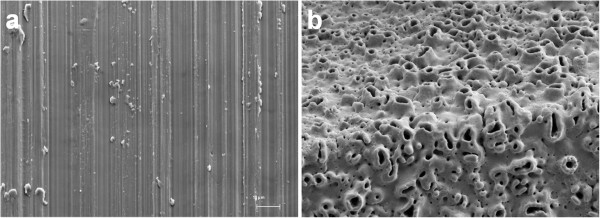
**Scanning electron microscopy (SEM) images of machined (a) and anodized (b) titanium surfaces, provided by Nobel Biocare AB.** The machined surface is relatively smooth with distinct orientation of the surface irregularities (anisotropic) while the anodized surface is rough with a homogenous structure (isotropic).

Discs of commercially pure titanium (diameter of 15 mm) with either a machined or anodized (TiUnite™) surface were used (see Figure 1). The discs with machined surface had a thickness of 1.0 mm and the discs with anodized surface had a thickness of 1.7 mm. All discs were produced, cleaned, sterilized and delivered in ethanol by Nobel Biocare AB. The average surface roughness (S_a_) of the discs was measured at Nobel Biocare AB after production, over a 88 × 76 μm area by interference microscopy using the Mapview EX surface mapping software and a Gaussian high-pass 50 × 50 μm filter. The S_a_ value (± standard deviation) was found to be 0.48 ± 0.04 μm for the machined surface and 1.26 ± 0.09 μm for the anodized surface, corresponding to values presented in the literature for equivalent implants [24,25]. The S_a_ value was obtained by calculating the arithmetic mean of the absolute values of the measured surface departures from a mean plane within the sampling area [26]. The Gaussian filter was applied in order to separate the roughness from the waviness and form of the surface [25]. The S_a_ was measured for at least 2 locations per triplicate of each surface.

### Preparation of inoculums

The early colonizers *S. mitis* (CCUG 27741) and *A. oris* (CCUG 33517), originally derived from human dental plaque (Culture Collection, University of Gothenburg, Sweden), were grown on horse blood agar plates. Overnight cultures of the two bacteria were incubated separately for 19 hours in brain heart infusion (BHI) medium, 37 g l^-1^ (Difco Laboratories, Detroit, Michigan, USA) supplemented with glucose, 10 g l^-1^, cysteine hydrochloride, 0.5 g l^-1^ (VWR International, Stockholm, Sweden ), yeast extract, 5 g l^-1^ (Difco Laboratories, Detroit, Michigan, USA) and inactivated horse serum 1% (Fisher Scientific, Göteborg, Sweden). The 50/50 co-culture (10^6^ CFU ml^-1^) of *S. mitis* and *A. oris*, in new supplemented BHI broth, was prepared directly prior to use. Agar plates and overnight cultures were incubated in an anaerobic jar at 37°C with CO_2_Gen (Oxoid, Malmö, Sweden) to create an atmosphere rich in carbon dioxide (CO_2,_ 6%) and poor in oxygen (O_2_, 15%). The additional serum and proteins where added to the medium to imitate the gingival crevice fluid and to form a protein layer on the titanium surfaces, like the pellicle formed on hard oral surfaces.

### Bacterial lag phase duration and initial adhesion

The lag phase duration (LPD) of *S. mitis* and *A. oris,* in mono and co-culture, was measured. Bacteria were cultivated in supplemented BHI broth, pH 7.0, at 37°C in a modified atmosphere (CO_2_, 6%; O_2_ 15%) as described above. Seven samples were withdrawn at intervals throughout a 4-hour period of incubation. Samples were diluted as appropriate and spread on agar plates for colony-forming unit (CFU) counting, after incubation at 37°C in an anaerobic jar for two days, as described above. The LPD was determined by fitting the model of Baranyi and Roberts [27] to the growth curves using the freeware MicroFit 1.0 (Institute of Food Research, Norwich, UK).

The initial adhesion of *S. mitis* and *A. oris,* in mono and co-culture, after 2.0 hours of incubation at pH 7.0 in modified atmosphere (CO_2_, 6%; O_2_ 15%) was measured. Ethanol-sterilized titanium discs with machined and anodized surface were placed in a 12-well microtiter plate and incubated in 2.0 ml of supplemented BHI broth (pH 7) inoculated with the two bacteria to a concentration of 10^6^ CFU ml^-1^ of each bacterium, in single as well as in co-culture. Incubation was performed on an orbital shaker (80 revolutions per minute, rpm) at 37°C. The measurements were repeated twice.

### Incubation conditions for bacterial adhesion

Supplemented BHI broth with a pH of 5.0, 7.0 or 9.0 was inoculated with *S. mitis* and *A. oris* to a concentration of 10^6^ CFU ml^-1^ of each bacterium. Machined and anodized titanium discs were placed in a 12-well microtiter plate as described above, covered by 2.0 ml of the inoculated BHI broth of different pH and incubated in aerobic or anaerobic atmosphere. Incubation was carried out at 37°C on an orbital shaker (80 rpm) for 2.0 hours. The experiments with all combinations of pH, atmosphere and surface were carried out at least six times, on separate occasions.

The values of pH (5.0 and 9.0) were chosen to represent the fluctuations in environmental pH that occur in the mouth depending on location in the dental plaque, food intake and oral health. The pH of whole saliva ranges from 6.75 to 7.25 [28], and pH 7.0 was therefore used as reference. The pH of the supplemented BHI broth was adjusted with HCl or NaOH prior to sterilization (at 121°C for 15 minutes) and also analysed after sterilization to detect if any deviation from the specified value had occurred. When stored aerobically for a minimum of 24 hours at 5°C the supplemented BHI broth, reached a saturation of dissolved oxygen of approximately 80%, which was used to represent the aerobic environment of the gingival margin. The oxygen deprived environment in the gingival crevice and mature plaque was simulated by flushing the broth with nitrogen for two minutes directly prior to use, to reduce the oxygen saturation in the broth from 80% to 20%. Incubation was performed in a sealed blender bag (VWR International, Stockholm, Sweden) equipped with a membrane through which the air was extracted with a syringe and replaced with nitrogen. This was done in order to prevent the oxygen saturation in the broth to increase during the extent of the experiment. The oxygen saturation of the BHI broth was measured at room-temperature with an oxygen meter (Oxi 340i) equipped with an oxygen gas electrode (CellOx 325) (both from WTW, Weilheim, Germany) at three separate occasions, prior to the experiment. The oxygen saturation of the BHI broth was found to decrease to 20% after 1 minute of nitrogen flushing and did not decrease further although flushed for up to 20 minutes. Thus, a time of 2 minutes was chosen for the experiments.

### Enumeration of adhered bacteria

After incubation, each titanium disc was rinsed in sterile distilled water for 10 seconds to remove non-adhered bacteria. The disc was then placed in a plastic blender bag (VWR International, Stockholm, Sweden) with 42 ml sterile buffered peptone water (0.85% NaCl and 1% Peptone) (Difco Laboratories, Becton Dickinson, Stockholm, Sweden) and processed in a stomacher as described by Gagnon and Slawson [29] with 500 strokes per minute for 60 seconds, in order to mechanically remove the adhered bacteria from the titanium disc.

The peptone water solution containing the detached bacteria was diluted 1:10, spread on blood agar plates and incubated as described above for CFU counting. The colonies formed by the two bacteria differ in colony-morphology, and in respect to size, colour and shape allowing for the CFU of the two bacteria to be counted separately. The total amount of bacteria on the discs after 2.0 hours of incubation could be calculated by counting the CFU on agar and divided by the area of the discs exposed to the bacteria. Since the disc was placed on the bottom of a microtiter well, only very small numbers of bacteria were found to adhere to the underside of the disc (microscopy analysis data not shown in proportion to the top-side and edges. Based on this, the bacteria that adhered to the bottom-side was deemed not to affect the overall comparison, and only the upper surface and the side of the disc were included when calculating the area. Although, the anodized surface has been suggested to have a 95% larger area than an ideal flat surface, [24] it was considered to be of more clinical relevance to calculate the area at mm level, not taking in to account the increase in surface area at μm level of the anodized surface due to the structured surface.

### Microscopy techniques

Titanium discs were incubated with the bacterial co-culture as described above , stained with 10 μl LIVE/DEAD® (1% in sterile distilled water) (Molecular Probes, Stockholm, Sweden) and incubated in the dark for 20 minutes. The bacterial adherence was analysed using confocal laser scanning microscopy (CLSM) with an inverted microscope (Leica DM IRE2, Leica Microsystems, Manheim, Germany) equipped with a glycerol immersion objective with a magnification of 63 times and a numerical aperture of 1.3. The fluorochromes indicating live and dead cells were excited by 488 and 594 nm light, respectively. The emitted light was collected in the wavelength ranges 500–554 and 620–660 nm, the former (green) indicating viable cells and the latter (red) non-viable cells. *S. mitis* and *A. oris* could be visually separated by their difference in cell- and colony-morphology (cocci and chains v.s. rod and clusters, respectively). Samples were prepared and analysed in duplicate on two separate occasions. The resolution of all images was 0.12 μm per pixel.

The amount of adhered bacteria on the bottom-side of the titanium discs after 2.0 hours of incubation was analyzed by staining the surfaces with LIVE/DEAD® as described above and imaging them with a fluorescence microscope (Axioskop, Carl Zeiss, Oberkochen, Germany). The surfaces were imaged after stomaching (1 min) to make sure that adhered bacteria were removed satisfactorily. This examination was performed on duplicates of each surface, one incubated at pH 5.0 and the other at pH 7.0, at 37°C and modified atmosphere (CO_2_, 6%; O_2_ 15%).

### Statistical analysis

Levene’s test of equality of error variances was used to establish that the logarithm (base 10) of the number of bacteria adhered to the machined and anodized titanium discs (log CFU mm^-2^) had a homogeneous variance. As this was shown to be the case, three-way analysis of variance (ANOVA) could be performed to statistically analyse the effect of pH 5.0 and 9.0 in comparison to pH 7.0 combined with aerobic or anaerobic atmosphere on the adhesion of *S. mitis* and *A. oris* in co-culture to machined and anodized titanium surfaces. The effects of surface, pH and atmosphere on the number of adhered *S. mitis* and *A. oris* (log CFU mm^-1^), in co-culture, were analysed. Dunett’s t-test was used to compare the effect of pH 5.0 and 9.0 versus pH 7.0. As the other factors (atmosphere and surface) only had two levels, no post hoc test was needed. All analysis was performed in SPSS Statistics version 17.0 and the criterion for statistical significance was set to α = 0.05 (i.e. comparisons rendering a p-value smaller than 0.05 are considered to be of statistical significance). The factors investigated were found to influence the adhesion of *S. mitis* and *A. oris* differently. However, since the bacteria were in a co-culture, they could not be considered to be independent of each other, and no statistical analysis of the difference between the two bacteria was performed.

## Results

### Bacterial lag phase duration and adhesion

The lag phase duration of *A. oris* was found to be 2.4 hours in both mono and co-culture (data not shown). The LPD of *S. mitis* was found to be 3.0 hours in monoculture and 2.8 hours in co-culture. After 3–3.5 hours, coinciding with the log phase of *S. mitis*, the lag phase of *A. oris* in co-culture was found to be disturbed, with a decrease in the number of viable cells as a result. Since the aim of the study was to investigate the initial bacterial adhesion and not the growth on the surface or the competition between the two bacteria in co-culture, an incubation time of 2.0 hours was chosen for the experiments.

The adhesion of *S. mitis* and *A. oris,* in co-culture, incubated for 2.0 hours at optimal conditions (pH 7.0, 37°C, CO_2_: 6%, O_2_: 15%) was measured. *S. mitis* was found to adhere to the machined and anodized titanium surfaces in amounts of 2.3 ± 0.14 and 2.0 ± .039 log CFU mm^-2^ , and *A. oris* in amounts of 1.9 ± 0.55 and 1.8 ± 0.26 log CFU mm^-2^, respectively. Since 2.0 hours of incubation was found to be sufficient for bacterial adhesion, the continuing experiments were performed with the same time of incubation, although at different pH and atmosphere.

### Effects of environmental factors on bacterial adhesion

**Figure 2:**
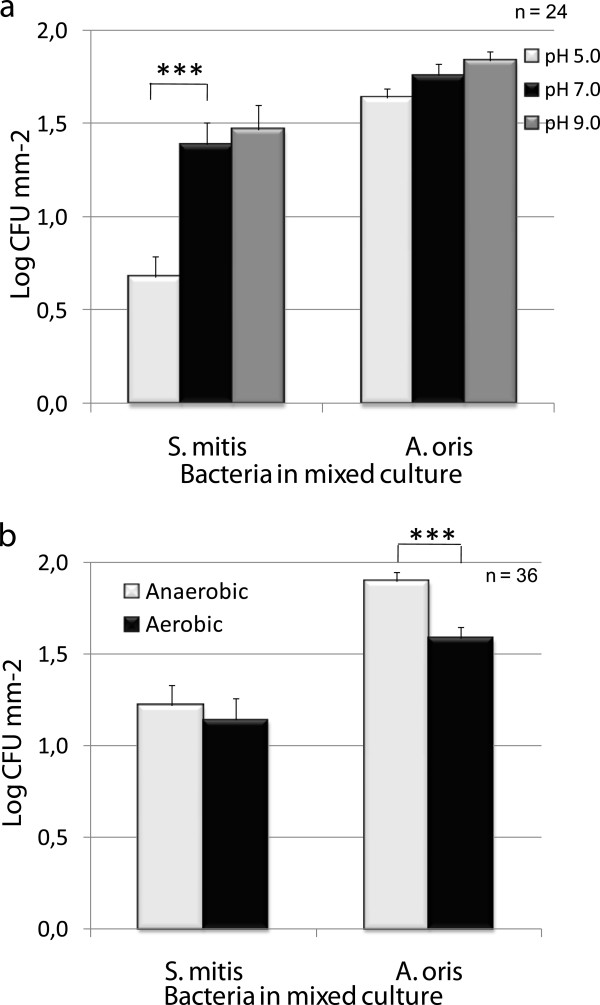
**Effects of environmental pH (5.0, 7.0 and 9.0) and aerobic or anaerobic conditions on the adhesion of *S. mitis *and *A. oris *in co-culture after 2.0 hours’ incubation.** Statistically significant less adhered *S. mitis* was found after incubation at pH 5.0 than pH 7.0 and of *A. oris* after incubation at aerobic environment compared to anaerobic environment.

The acidic pH of 5.0 was found to reduce the adhesion of *S. mitis* to titanium surfaces by 50% when compared to pH 7.0 (see Figure 2), whereas pH had no effect on the adherence of *A. oris*. Furthermore, the adhesion of both bacteria was found to be unaffected by incubation at pH 9.0 when compared to pH 7.0.

**Figure 3:**
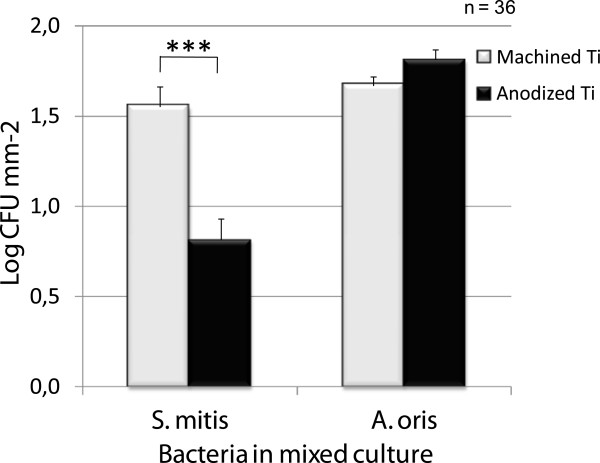
**Effect of surface properties on the adhesion of *S. mitis *and *A. oris *in co-culture to titanium after 2.0 hours of incubation.** Statistically significant less adhered *S. mitis* was found on the anodized surface than the machined titanium surface.

Figure 2b shows that the amount of *A. oris* adhered to the two titanium surfaces were found to be significantly higher after incubation in an anaerobic environment than in an aerobic environment. The adhesion of *S. mitis,* on the other hand, was found to be unaffected by the aerobic atmosphere, and was found in equal amounts after incubation in both anaerobic and aerobic environments. The amount of *S. mitis* found on the anodized surface was half of that found on the machined surface (see Figure 3), which was of statistical significance. *A. oris* was found to adhere in equal amounts on both surfaces. The exact numbers for the adhesion as affected by the main factors are presented in Table 1.

**Table 1:** **The mean number of adhered bacteria (log CFU per mm**^**2**^**± SEM) to the titanium disc, as affected by surface, pH and atmosphere, are presented for each of *S. mitis *and *A. oris***

	**Surface**		**pH**			**Atmosphere**	
**Bacteria**	**Machined Ti**	**Anodized Ti**	**5.0**	**7.0**	**9.0**	**Anaerobic**	**Aerobic**
**S. *mitis***	**1.55 ± 0.12**	**0.81 ± 0.07**	**0.68 ± 0.08**	**1.39 ± 0.14**	**1.47 ± 0.13**	**1.22 ± 0.11**	**1.14 ± 0.12**
**p**	0.001*		0.001*			**0.483**	
**A. *oris***	**1.67 ± 0.07**	**1.81 ± 0.05**	**1.64 ± 0.08**	**1.76 ± 0.08**	**1.84 ± 0.052**	**1.90 ± 0.05**	**1.59 ± 0.06**
**p**	**0.065**		**1.106**			0.001*	

Discs are incubated with the bacteria in co-culture however CFU for the two bacteria are separated by their difference in colony morphology. The number of CFU as affected by surface, pH and atmosphere was analyzed with ANOVA and the results for the main factors presented in this table. The p-values indicating significant difference are marked with *.

**Figure 4:**
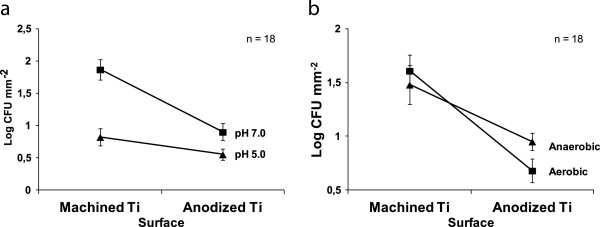
**Interactions between surface, pH and atmosphere affects tha bacterial adhesion.** The first figure (**a**) illustrates the effect of pH and surface properties on the adhesion of *S. mitis* in co-culture with *A. oris.* The adhesion of *S. mitis* is less on both surfaces after incubation at pH 5.0 than 7.0 the reduction is however statistically significant larger for the adhesion to machined titanium than anodized titanium. Figure (**b**) illustrates the effect of surface properties and aerobic or anaerobic environment on the adhesion of *S. mitis* in co-culture with *A. oris*. The adhesion to the anodized surface was found to be reduced by aerobic incubation while the opposite was found for the adherence to machined titanium, this difference is however not statistically significant.

In addition, important (and statistically significant) interaction effects were found between pH and surface. The adhesion of *S. mitis,* in co-culture with *A. oris*, is reduced on both surfaces after incubation at pH 5.0 compared to incubation at pH 7.0. This reduction is statistically more significant for the adhesion to machined titanium than anodized titanium, which had a low adherence of *S. mitis* also at pH 7, as shown by Figure 4a. Moreover, a borderline effect was found for the interaction between atmosphere and surface as shown by Figure 4b. The already low amount of *S. mitis* found on the anodized surface was further reduced by incubation in aerobic conditions while no difference in the adhesion to machined titanium was found dependent on atmosphere.

### Microscopy analysis

From the visual examination of the bottom-side of the titanium discs after 2.0 hours’ incubation, it was clear that some bacteria adhered to this side. However, this amount was small in comparison to that on the upper-side, which explains the exclusion of the bottom-side from the analysis. The same result, with a low number of remaining bacteria, was found when analysing the upper-side of the discs after stomaching (data not shown). These results confirm that 1 minute of stomaching is enough to remove adhered cells from the anodized surface as well as the machined. Furthermore, the number of remaining bacteria on the surfaces after stomaching was very low in comparison to the number of removed bacteria and was, therefore, deemed not to have effect on the statistical analysis.

**Figure 5:**
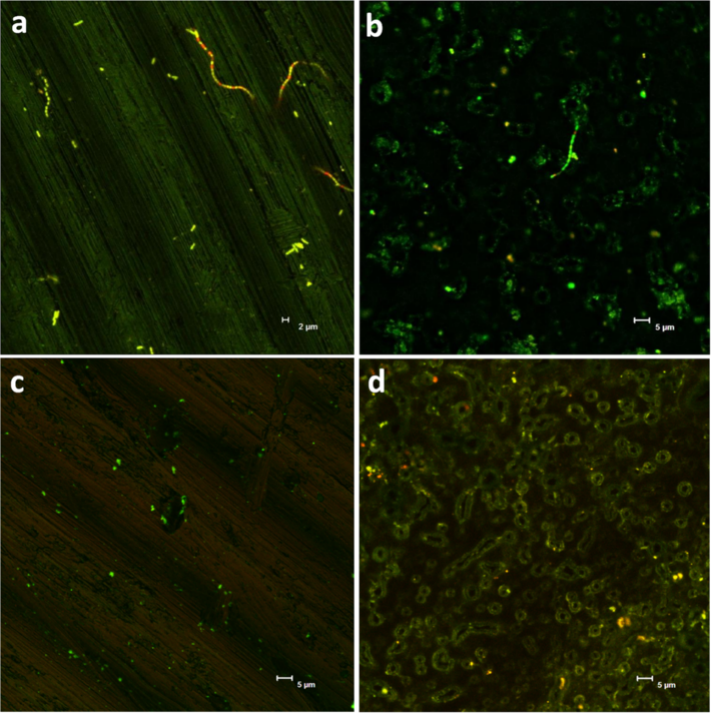
**Confocal laser scanning microscopy images of machined titanium (a,c) and anodized titanium (b,d) surfaces incubated with a co-culture of *A. oris *and *S. mitis.*** The bacteria are labeled with LIVE/DEAD® (Molecular Probes) thus green color indicates live cells and red color indicates dead cells. Surfaces a-c were incubated anaerobic and d aerobic. The two bacteria were found in equal amounts on the machined titanium surface after anaerobic incubation at pH 7 (**a**) while mostly *A. oris* could be found on the anodized surface, after incubation at the same conditions (**b**). The adhesion of *S. mitis* to both surfaces was further reduced after incubation at pH 5 irrespective of atmosphere (**c**,**d**). The number of non-viable cells (red) of *A.oris* was found to be greater after aerobic incubation than anaerobic (**d**).

**Figure 6:**
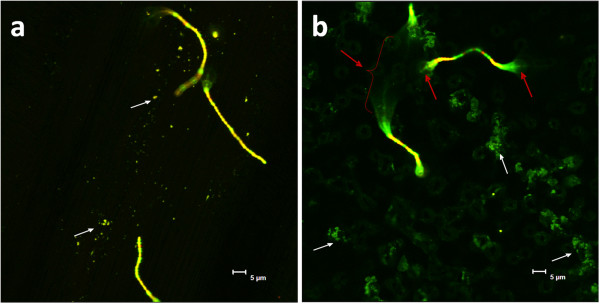
**Confocal laser scanning microscopy images of *A. oris *(small distinct dots and clusters, indicated by white arrows) and *S. mitis *(chains) adhered to machined (a) and anodized (b) titanium surfaces, after aerobic incubation at pH 7.** The bacteria are labelled with LIVE/DEAD® (Molecular Probes) visualizing live cells in green and dead cells in read. Red arrows indicate partly detached chains of *S. mitis* .

CLSM was performed to obtain more information on the bacterial adhesion to the surfaces and the viability of attached bacteria. *A. oris* was often found as single cells or in small clusters, adhering to the grooves formed by the machining process of the machined surface; and around the structures of the anodized surface, as seen in Figure 5. *S. mitis* was also found to adhere to the grooves of the machined titanium surface (Figure 6a) while the structure of the anodized surface seemed to obstruct the adhesion of this bacterium since the chains were often found partly detached from the surface (Figure 6b). The surfaces to the left in Figure 5 are machined titanium while the right surfaces are anodized titanium. Moreover, live cells are visualized in green and dead cells are seen in red. Surfaces a-c were incubated in anaerobic atmosphere, surface d in aerobic atmosphere. Surfaces c-d were incubated at pH 5. In line with the above described results, higher amounts of the long chains formed by *S. mitis* were found on the machined surface than on the anodized surface, as illustrated by Figure 5a-b. Equal amounts of the two bacteria were found on the machined surface after anaerobic incubation at pH 7 (Figure 5a) while the adhesion of *S. mitis* to both surfaces was reduced by incubation at pH 5 irrespective of atmosphere (Figure 5c-d). A high viability of both bacteria was seen after incubation at pH 7.0, with only few non-viable cells in the clusters and chains. The amount of non-viable cells of *S. mitis* was, however, greater after incubation at pH 5.0 (data not shown) and the same was found for *A. oris* after incubation at aerobic environment (Figure 5d).

## Discussion

Our results show that the initial adherence of *S. mitis* to titanium is significantly reduced by incubation at pH 5.0, which is in line with results presented in the literature for the adhesion of *S. sanguinis* to hydroxyapatite [30,31]. These findings are interesting, and the potential of a short acidic treatment inducing bacterial dispersal is something worth further investigation, using more mixed species cultures as well as in clinical investigations.

No limiting effect of pH 5.0 could be seen on the initial adhesion of *A. oris* to titanium in the present study. Horiuchi *et al*. [32] have shown that 35-76% of *A. oris* in planctonic culture died after acidification at pH 4.0 for 1 hour and Svensäter *et al.*[33] found no surviving bacteria after 3 hours incubation at pH 3.2 . This indicates that *A. oris* is sensitive to an acidic environment when grown in continuous culture, but that a pH of 5.0 is not low enough to harm attached bacteria or inhibit adhesion. It is known that, when associated to a surface [11,34] or in a biofilm [35,36] bacteria are more tolerant to surrounding stress factors, such as antimicrobial agents or toxic environment. In fact, stress factors may lead to increased adhesion and biofilm formation [37-39]. Additional work is needed in order to define the border line between a pH initiating moderate stress leading to increased adhesion and a pH acidic enough to inhibit adherence/initiate dispersal or killing.

A tendency toward a higher amount of adhered bacteria on the two surfaces was seen after incubation at pH 9.0, indicating a tolerance among oral bacteria to alkaline environments. This is in accordance to results presented for root canal bacteria [40]. Apical periodontitis is commonly treated with mechanical instrumentation combined with disinfectants, such as intra-canal calcium hydroxide dressings. Although direct contact with this alkaline chemical should be lethal, alkaline-tolerant organisms are often found in the microflora after treatment. This indicates that the survival mechanisms triggered by alkaline stress could be part of a general adaptive survival response in oral bacteria. Chávez de Paz et al. further noted that organisms usually preferring an acidic environment (e.g. some *Streptococcus, Lactobacillus* and *Fusobacterium nucleatum*) survived, and were able to adapt to alkaline stress at pH 10.5 for four hours [40].

In the present study, *A. oris* was found to adhere equally well to both the rough anodized and smooth machined titanium surfaces. This is in contradiction to our hypothesis that the anatase layer of the rough surface would prevent bacterial adhesion. It is suggested that the antimicrobial properties of the anatase layer increase after irradiation with ultraviolet (UV) light [41]. Therefore, additional experiments are needed in order to establish if UV-treatment will increase the antimicrobial properties of the investigated anodized surface, as has been shown to be the case for other titanium oxide surfaces [42]. The adherence of *S. mitis,* however, was found to be significantly less on the anodized surface than on the machined titanium surface, and even further reduced by aerobic incubation. When analysed by CLSM, the adherence of *S. mitis* was frequently found to be limited to the crests of the structures while the sections of the chain in between where floating free in the medium (seen as unfocused cells in Figure 6b, indicated by arrows) which is a sign of poor adhesion. On the machined surface, however, most cells in the chains of *S. mitis* had surface contact (Figure 6a) promoting a strong adhesion. These results indicate that the surface topography could be more important than the anatase layer in restricting the adhesion of this bacterium. This observation coincides with earlier results showing a decrease in the adhesion of *S. mitis* after roughening of the denture surface [43], although contradictory results, with more adhesion on rough surfaces, been observed in *in vitro* investigations using other *Streptococci* as model bacteria for surface attachment [44,45] stressing the need of including more species to the consortium. Moreover, *in vivo* studies on biofilm formation have shown more adhesion on rough surfaces as well as a clear influence of surface free energy when placed in supragingival locations [44,46]. Subgingivally, however, the biofilm formation is more dependent on the oral health of the patient than the nature of the surface [46,47]. The complexity of biofilm formation on customized titanium surfaces is further visualized by the *in sit*u study of Fröjd et al. (2011) [48] where anodization of the titanium surface was found to promote biofilm formation. These opposing results are hard to interpret, as there are fundamental differences in performance, surface characteristics and production of the anodized titanium surface; as well as variations in the methods used to quantify the bacterial load. Nevertheless, these opposing results emphasize the importance in these experiments of carefully designing the mixed bacterial consortia as well as using both *in vitro* and *in situ* analytical methods to evaluate the attachment of bacteria and biofilm formation on dental implants.

The complexity of biofilm formation was also demonstrated by the interaction effects, which show that it is not possible to extrapolate results from one condition to another. The results show that a higher amount of *S. mitis* adhere to the machined titanium surface than the anodized surface after incubation at pH 7.0 but that the reduction in adhesion after incubation at pH 5.0 compared to pH 7.0 is larger for the machined titanium surface than the anodized surface. This indicates that an acidic pH can inhibit adhesion of large amounts of bacteria but that it is not enough to inhibit bacterial adhesion in total. Anaerobic conditions were found to promote initial adhesion of *A. oris* whereas *S. mitis* was found to adhere in equal amounts during both aerobic and anaerobic incubation. However, a trend towards reduced adhesion on the anodized surface after aerobic incubation was found, suggesting that adhesion is affected when challenged both with aerobic environment and rough surface structure.

Although both *A. oris* and *S. mitis* are early colonizers found in similar oral environments, these two bacteria were found to respond differently to the tested factors. This strongly emphasises the importance of using several bacterial species for *in vitro* as well as *in situ* studies and, preferably, more than one strain of each species.

## Conclusions

The adhesion of *S. mitis* to titanium was restricted by an acidic environment and the anodized surface. The adhesion of *A. oris*, on the other hand, was reduced by aerobic incubation. These findings suggest bacterial adhesion to be sensitive to environmental changes, indicating a possibility to restrict bacterial adhesion after implant placement in order to promote healing. However, more bacterial species and longer incubation times need to be investigated. At the same time, complementary work should be performed on the effect of changes in environmental conditions on the dispersal of adhered bacteria. If the strength in adhesion can be reduced by altering pH before mechanical instrumentation, this could be a future way to establish implant surfaces that are free from bacteria and new ways of treating infections around implants.

## Abbreviations

BHI: Brain heart infusion; CFU: Colony forming units; CLSM: Confocal laser scanning microscopy; LPD: Lag phase duration; S_a_: Surface roughness; UV: Ultraviolet.

## Competing interests

JSC and JF are employed by Nobel Biocare.

## Authors’ contributions

JSC participated in planning the study and the experimental design, performed the laboratory work, the CLSM analysis and the statistical analysis and participated in drafting the manuscript. MLV, JF and ML participated in the study planning, experimental design, data analysis and drafting of the manuscript. All authors have read and approved the final manuscript.

## Pre-publication history

The pre-publication history for this paper can be accessed here:

http://www.biomedcentral.com/1472-6831/13/4/prepub
